# Challenges and Facilitators to Patient and Public Involvement In Stroke Research: Protocol for a Qualitative Study

**DOI:** 10.1111/hex.70231

**Published:** 2025-04-01

**Authors:** Hamidreza Khankeh, Shima Shirozhan, Ulrich Dirnagl

**Affiliations:** ^1^ QUEST Center for Responsible Research, Berlin Institute of Health at Charité, Berlin, Germany. Health in Emergency and Disaster Research Center, Social Health Research Institute University of Social Welfare and Rehabilitation Sciences Tehran Iran; ^2^ Department of Nursing, Health in Emergency and Disaster Research Center, Social Health Research Institute University of Social Welfare and Rehabilitation Sciences Tehran Iran; ^3^ QUEST Center for Responsible Research Berlin Institute of Health at Charité Berlin Germany

**Keywords:** patient Advocacy, patient and public involvement, qualitative research, stakeholder engagement, stroke

## Abstract

**Background:**

Patient and public involvement in the research process, despite its novelty and importance in enhancing the quality of studies, still has many unknown dimensions that need to be discovered and explained through qualitative research. Conducting qualitative research to understand experiences in newly emerging topics is challenging and can pose significant difficulties for researchers.

**Objective:**

Development of a directed qualitative content analysis study protocol to identify challenges and facilitators to patient and public involvement in stroke research.

**Design:**

Study protocol of qualitative content analysis.

**Method:**

This study protocol outlines the step‐by‐step design for a directed qualitative content analysis. The study will use a directed approach, following the methodology proposed by Hsieh and Shannon (2005). Participants will be selected based on their experience or knowledge of involvement in the research process, including stakeholders such as researchers, people who are affected by stroke, caregivers, and healthcare providers in stroke research. Participants will be purposefully chosen for their willingness and ability to contribute. Data will be gathered through semi‐structured in‐depth interviews. The quality of the study will be ensured by applying the criteria established by Lincoln and Guba.

**Conclusion:**

This study protocol offers a comprehensive guide for conducting directed qualitative content analysis, outlining the research process step‐by‐step to aid researchers using similar methodologies. It addresses common language‐related challenges and suggests solutions. The protocol emphasizes maintaining high research standards through specific criteria and provides a detailed discussion of ethical considerations. The authors advocate for publishing qualitative research protocols before implementation to improve research quality, foster ethical and integrated practices, and support novice researchers.

**Patient or Public Contribution:**

Stakeholder involvement was crucial in developing the interview guide. Feedback was gathered from six experts: three researchers (quantitative and qualitative fields), one stroke rehabilitation specialist, and two Patient and Public Involvement (PPI) experts. Their insights guided the research team in determining research areas, proposing participant recruitment strategies, addressing language barriers, and ensuring necessary details were provided for the study protocol.

**Abbreviations**
PPI: Patient and Public Involvement
PSE: Patient and Stakeholder Engagement
SPSE: Stroke Patient and Stakeholder Engagement

## 1 Background

Incorporating people into clinical research through patient and public involvement (PPI), also known as Patient and Stakeholder Engagement (PSE), ensures patient‐centred studies, enhancing feasibility, credibility, and ethical conduct [1, 2, 3]. This approach leads to more appropriate outcomes and improved trust and communication between researchers and the patient community [4, 5]. Additionally, it raises the overall quality of research studies [3, 4, 6, 7].

One of the most important areas that can benefit from PPI in the research process approach is stroke research. Given that stroke is the second leading cause of death and long‐term disability worldwide, and its prevalence is increasing among aging populations, it is crucial to emphasize the importance of addressing this condition [8, 9, 10] Also, optimal stroke prevention and management require collaboration among governments, scientific organizations, healthcare professionals, researchers, patients, and their families [8, 11]. In this regard, the European Stroke Action Plan underscores the need to connect research findings with patient communities and stakeholders [12].

It is important to note that despite the numerous benefits of the PPI approach and the significance of conducting quality research in stroke management, this approach has received little attention in the field of stroke. The scope of PPI in stroke research remains unclear, necessitating comprehensive studies to identify barriers, challenges, gaps, needs, and opportunities, as well as to provide applied recommendations regarding PPI [5, 13, 14, 15].

Based on the need for this research gap, a scoping review study has been published by this research team with the aim of examining existing evidence and research gaps, investigating the concepts, definitions, models, implementation strategies, indicators, challenges, facilitators, principles, and frameworks of Stroke Patients and Stakeholders Engagement (SPSE) [16].

Considering the findings of the review study and other research, PPI in the research process should begin from the study's design and extend through all stages until the dissemination of results [5, 13, 16, 17]. At each stage, researchers should determine the method and level of involvement, though this process is complex and associated with unknowns [2, 15, 16, 18, 19]. Researchers may be unfamiliar with the research journey and should actively involve stakeholders, adhering to ethical considerations and principles throughout [2, 4, 5, 13, 15, 16, 17, 18, 19, 20].

Moreover, the stakeholders are diverse, each member with unique characteristics, needs, values, and concerns [5, 15, 16, 20]. For instance, people who are affected by stroke may face communication challenges due to speech or cognitive impairments, requiring researchers to choose appropriate communication methods. These challenges have been discussed in previous studies, which often focus more on the challenges than solutions and lack a connection between challenges and strategies [16, 18, 19].

Understanding the experiences and perceptions of stroke survivors and their stakeholders provides an opportunity to identify real challenges rather than anticipated ones [5, 13, 14, 15, 16]. Given the complexity and unfamiliarity of the PPI process, qualitative research is appropriate and can help researchers deeply understand participants' perspectives and experiences. Only with a deep understanding of these phenomena can researchers find solutions and recommendations to overcome unpredictable challenges and issues [2, 4, 5, 13, 15, 17, 18, 19, 20].

Our vision is to provide a standardized, research‐based, and stakeholder‐comprehensive approach to PPI, fostering collaboration among researchers, patients, and stakeholders. The evidence‐informed recommendations developed will support PPI activities at other institutions, aligning with the ‘Rethinking Health‐Charité 2030’ strategy, which aims to advance health, care, and research as an international driving force [21].

Recognizing the explorative nature and complexity of qualitative research, we designed the study and developed a comprehensive study protocol, presented in this article. We believe that this protocol will enhance the accuracy and quality of implementation, increase transparency and integrity, and provide valuable insights for other researchers in designing and conducting similar studies.

This protocol will attempt to guide the conduct of a qualitative study aimed at gaining an in‐depth understanding of challenges and facilitators to PPI in stroke research. It is designed to complement the findings of our systematic scoping review, which recognized various types of evidence related to SPSE, summarized experiences and recommendations, clarified key concepts, definitions, and components, and outlined models, implementation strategies, indicators, and frameworks for launching SPSE.

### 1.1 Aim

Development of a directed qualitative content analysis study protocol to identify challenges and facilitators to PPI in stroke research.

### 1.2 Research Questions

1. What are the key challenges to implementing PPI in stroke research in Germany as a high‐income country?
2. What factors facilitate the successful integration of PPI in the design, conduct, and dissemination of stroke research in Germany as a high‐income country?
3. How do researchers, people who are affected by stroke, caregivers, and healthcare providers perceive their roles in PPI within stroke research?
4. What factors can encourage stakeholders to involve in the research process?

## 2 Anticipated Duration of the Study

The Stroke Patients and Stakeholders Engagement (SPSE) project runs from May 2024 through May 2026. Interviews are planned to take place from May 2025 through September 2025.

### 2.1 Methodology/Study Design

This study is a qualitative‐directed content analysis study protocol that will be conducted at the Berlin Institute of Health at Charité (BIH), QUEST Center for Responsible Research, in Germany as a high‐income country. This project was supported by the Deutsche Forschungsgemeinschaft (DFG) under Project number 539333509, dated November 21, 2023. Based on existing research and the identified knowledge (as referenced in our scoping review of SPSE [22]), this directed content analysis will follow the approach proposed by Hsieh and Shannon [23]. To conduct this protocol, we utilized the GRIPP2 checklist for PPI, along with the ObsQual checklist qualitative study protocols [24, 25].

### 2.2 Field of the Study

The setting of this study encompasses various locations where qualitative data will be collected, focusing on participants who have experience with stroke. Given the diverse range of stakeholders involved in stroke research, the study will be conducted in several key locations in Berlin, including Hospitals, Outpatient Clinics, Rehabilitation Centres, Research and Educational Centres, and Homes of people who are affected by stroke.

### 2.3 Research Participants and Selection Criteria

We will start with purposive sampling and to ensure maximum diversity in participant selection, we will use snowball sampling as a complementary strategy if needed for data collection [26]. We will start with purposive sampling to include a range of perspectives across stroke research including people who are affected by stroke, family members as lay caregivers, healthcare providers, and researchers. We will ask related organizations, such as the Berlin Stroke Alliance (BSA e.V.), to assist us in finding volunteers among stroke survivors, their family caregivers, and healthcare providers for recruitment in the study. Additionally, we will enquire if interviewees can recommend other individuals. This snowballing approach aims to close knowledge gaps around our research questions by including perspectives we may not have previously considered or could obtain through purposive sampling.

We aim to conduct approximately 20 interviews, with around five participants per category, focusing on individuals knowledgeable and experienced in stroke research. The final number of interviews will depend on data saturation [26]. Following a directed qualitative content analysis approach based on our systematic scoping review, we start with predefined concepts, categories and frameworks, generally requiring fewer participants compared to inductive analysis. We believe a minimum of five participants per stakeholder group will capture key perspectives, but we are open to increasing the sample size if needed to achieve saturation.

#### 2.3.1 Inclusion Criteria

The inclusion criteria for the study, categorized by stakeholder group, along with the exclusion criteria, are listed in Table 1.

**Table 1:** Inclusion and exclusion criteria for study participants by stakeholder group.

Inclusion criteria	Exclusion criteria
**General criteria for all participants:** ▪ Experience in Patient and Public Involvement (PPI) in stroke research ▪ Willing to share experiences, knowledge, and perceptions related to PPI and participate to study ▪ Ability for effective communication in Farsi, English, or German ▪ Provide informed consent	▪ Unwillingness to continue participation in the study ▪ Change in the patient's clinical condition to an acute state
**People who are affected by stroke:** ▪ Diagnosed with stroke and living in Berlin ▪ Introduced through relevant organizations (e.g., Berlin Stroke Alliance)	
**Family members/family caregivers of people who are affected by stroke:** ▪ Directly involved in the care or support of people who are affected by stroke	
**Healthcare providers:** ▪ Active involvement in the care of people who are affected by stroke (e.g., physicians, nurses, therapists, rehabilitation specialists) ▪ Currently working in a hospital or healthcare facility in Berlin or affiliated with organizations such as BSA e.V.	
**Researchers:** ▪ Actively conducting research in the field of stroke management, rehabilitation, or SPSE‐related fields. ▪ Experience with SPSE designing, conducting, and/or evaluation. ▪ Affiliated with healthcare facilities, research institutions or universities in Berlin.	

#### 2.3.2 Participant Selection

Diversity in participant characteristics will be prioritized, including:

- **Different Work and Research Backgrounds:** Ensuring a range of professional experiences among healthcare providers and researchers.
- **Research Paradigms:** Including participants with experience in quantitative, qualitative, and mixed methods research.
- **Demographic Diversity:** Considering different ages, education levels, and backgrounds for patients, family members, caregivers, and healthcare team members.

This approach will support to ensure a comprehensive understanding of the various perspectives on the challenges and facilitators to PPI in stroke research.

#### 2.3.3 Study Limitation

The primary limitation of this study, conducted in Germany, is the language barrier, a common challenge in cross‐language qualitative research. As the principal investigator is not a native German speaker, he cannot independently conduct interviews. To manage this, the research team will prioritize recruiting individuals proficient in Farsi or English and conduct interviews in English with proficient speakers. For German‐speaking participants, a native German‐speaking member of the research team will conduct the interviews and translate all transcriptions into English. The principal investigator will provide training to ensure consistency. The majority of interviews will be conducted in English and Farsi.

### 2.4 Data Gathering/Synthesis Strategies

Data collection for this study will be conducted using semi‐structured in‐depth interviews. We will contact all potential interviewees via email or telephone, providing them with the study information, an overview of the main interview themes, and the consent form. Individual interviews will be conducted by the principal investigator or a translator/interpreter, either online or face‐to‐face, following the interview guide. The interviews will take between 45 and 60 min. They will be audio‐recorded, transcribed, and pseudonymized after proofreading. Audio recordings will be erased once transcription is successfully completed.

### 2.5 Semi‐Structured Interviews

#### 2.5.1 Interview Guide Development

◦ The interview guide has been developed based on the findings of the scoping review of SPSE.
◦ It will be provided in three languages including Farsi, English, and German.
◦ A few pilot interviews will be conducted in Berlin to evaluate and refine the formulated questions, with at least one pilot interview per category. Based on feedback from these pilot interviews, the guide will be refined. Further refinements may occur during the study using an iterative approach, depending on the data collected and analyzed. Any major modifications will be versioned and accompanied by a justification (explaining why the changes were relevant or necessary). The interview guide will be publicly accessible after all interviews are completed.
◦ Specific questions will be designed for each group of stakeholders, ensuring relevance to their experiences and roles of the study participants. The sample interview questions for different groups of study participants are presented in Table 2.

**Table 2:** Sample of main interview questions for different groups of study participants.

Study participants	Interview questions
Patients	‐ Please share your experience in participating in research. ‐ What information would you like the researcher to provide at the beginning of the research process?
Family/caregivers	‐ Please share your experience in participating in research. ‐ What should the researcher explain to you before starting the research?
Researchers	‐ Please share your experience of engaging with stakeholders. ‐ How do you ensure the quality of studies conducted with stakeholder engagement?
Healthcare providers	‐ What suggestions would you provide to researchers for improving the research process? ‐ What conditions do you need in your workplace to engage in research?

#### 2.5.2 Conducting Interviews

◦ Before the interview begins, the purpose of the study and an explanation of the project will be provided to the participant. Informed consent to participate in the study and to be audio recorded will be obtained in written or oral form.
◦ Interviews will start with introductory and warm‐up questions to establish rapport.
◦ Main questions will be developed based on the results of the scoping review and will pose to explore key topics, with probing and follow‐up questions used to delve deeper into responses.
◦ Interviews will be scheduled at convenient times and locations for participants, following approval from the Charité Ethics Committee, Berlin, Germany.

Data collection and interviews will continue until data saturation is achieved. This means that the main challenges and recommendations for involvement of people who are affected by stroke and their stakeholders in the research process have been fully identified, concepts have been sufficiently enriched, and no new concepts or insights are emerging. At this point, it is determined that the data has reached adequate depth and comprehensiveness [26]. The research team will meet regularly (at least every 2 or 3 weeks) to review interviews and discuss the study process and results.

### 2.6 Data Analysis

Interviews will be transcribed by the principal investigator, and the transcripts will be manually pseudonymized by removing names and any other directly or indirectly identifying information. Directed content analysis, as recommended by Hsieh and Shannon (2005), will be used to interpret meaning from content based on pre‐existing knowledge [23]. Here's the process for conducting data analysis using directed content analysis.

### 2.7 Preparation of Theoretical Foundation

**Identify Key Concepts/Theories:** Start by identifying a theoretical framework or key concepts from prior research (scoping review of SPSE [16]) to guide the analysis and create initial categories. Extracted initial categories and sub‐categories for directed content analysis, presented in Table 3, will guide researchers during data collection and analysis. Also, researchers have developed operational definitions for each coding category based on the scoping review results, ensuring clear guidelines on how each code should be consistently applied to the data (Table 4).

**Table 3:** Categories and sub‐categories for directed content analysis (extracted from previous studies^a^).

Category	Subcategory
Challenges of PPI in research process	Challenges related to research design Challenges related to research implementation Challenges related to research results dissemination Challenges related to stakeholders Challenges related to the nature of involvement Challenges related to the researcher
Recommendations for PPI	Recommendations for research design Recommendations for research implementation Recommendations for dissemination of findings
Principals in PPI	Basic principles Selection of research topic Selection of stakeholders Development of research plan Data collection and implementation Data analysis Dissemination of research findings Ethics
Facilitator of PPI	Facilitating stakeholder roles in PPI Facilitating the main researcher's performance in PPI Facilitating the research process in PPI

^a^ Scoping review of evidence related to SPSE [16].

**Table 4:** Operational definition of basic concepts of SPSE in the research process.

Concept	Definition
Barriers	The circumstances that counter SPSEs' ability to reach their intended goals or that promote unintended effects. For our purposes, it refers to contextual or individual factors that inhibit the effective implementation of SPSEs.
Challenges	The occurrence of issues, such as communication challenges, may arise during the research process, and overcoming these challenges requires effort, time, and skills such as problem‐solving.
Facilitators	The circumstances that help SPSEs to reach their intended goals. For our purposes, it refers to external or procedural factors that support the effective implementation of SPSEs. “Effective” in this context implies that the intended objectives surpass any potential adverse outcomes.
Principles	The principles of SPSE are the fundamentals that distinguish research with stakeholder engagement from research in which individuals have only participated. The engagement of stakeholders in decision‐making during the design of research, as well as their active participation in the implementation of a study and the publication of findings, are key principles of SPSE.
Strategies/practices	Measures (such as forming virtual groups or creating a special communication channel), through which the researcher engages the stakeholders in the research process.
Benefits	Positive results, such as improving the quality of research, that SPSE in the research process brings.
Outcomes	The consequences of engaging stakeholders in the research process can range from positive (including benefits such as improving the quality of research), to neutral, or negative (such as the burnout of the healthcare providers).

### 2.8 Data Review and Coding

- **Apply Initial Codes**: Review the data and apply the predetermined codes to relevant content. Data that fit the predetermined categories are coded accordingly.
- **Identify Uncoded Data**: As we apply the codes, we may come across data that do not fit the initial categories. This is expected and represents the potential for discovering new categories.

### 2.9 Refining Codes and Identifying New Categories

- **Create New Codes**: If some data do not fit the initial categories, we will create new codes to capture these concepts. This ensures that the analysis remains flexible and responsive to the data.
- **Revise Categories**: As new categories emerge, we will revisit the initial coding scheme and refine it to incorporate these new findings.

### 2.10 Analysis and Interpretation

- **Summarize Findings**: After coding the data, we will summarize the key categories, both from the pre‐established knowledge provided by the scoping review and any new insights that emerged during the analysis.
- **Compare with results of the scoping review**: Compare the findings with the result of the scoping review. Determine whether the data supports the explored knowledge regarding PPI from the scoping review, extend it, or suggest modifications.
- **Interpret New categories**: For any new categories that emerge, analyze their relevance to the research question and how they contribute to understanding the phenomenon under study.

### 2.11 Report Findings

- **Present the Analysis**: In the final report, we will present the findings by discussing both the categories from the results of the scoping review and the emergent categories.
- **Provide Evidence**: Use direct quotes or examples from the data to illustrate how the content fits into each category and subcategories.
  This process ensures that directed content analysis remains both theory‐driven and flexible enough to capture new insights. By following these steps, the analysis will yield rich insights into the challenges and facilitators to PPI in stroke research.
- **Preprint and publish results** in peer‐reviewed journals and present at international conferences to reach a broad audience.

## 3 Trustworthiness/Rigour

To ensure the trustworthiness and rigor of this study, we will follow the criteria outlined by Lincoln and Guba (1985), which include credibility, transferability, dependability, and confirmability [26, 27]. Additionally, we will incorporate perspectives from other scholars who have contributed to the understanding of trustworthiness in qualitative research.

### 3.1 Credibility

- **Definition:** Confidence in the ‘truth’ of the findings.

### 3.2 Strategies

◦ Triangulation: Using multiple data sources and settings.
◦ Member Checking: Participants will review the findings to ensure accuracy and resonance with their experiences.
◦ Peer Debriefing: Colleagues will examine and discuss the data and findings to offer objective feedback. The research team will convene every 2 to 3 weeks to review the study findings and research process.
◦ Prolonged Engagement: Investing ample time in the research setting to achieve an in‐depth understanding of the context.
◦ Thick Description: Providing detailed descriptions of the context, participants, and research process to convey the findings vividly.
◦ Reflexivity: Researchers will reflect on their own biases and how these may influence the research process and outcomes.
◦ Saturation: Data collection will continue until no new categories or insights emerge [26, 27, 28].

### 3.3 Transferability

- **Definition:** Showing that the findings have applicability in other contexts.

### 3.4 Strategies

◦ Providing a clear and detailed description of the research context, participants, and procedures to enable readers to assess the applicability of the findings to other settings [26, 27, 28].

### 3.5 Dependability

- **Definition:** Showing that the findings are consistent and could be repeated.

### 3.6 Strategies

◦ Ensuring a systematic and transparent research process that can be followed by others.
◦ External Audits: Having an independent researcher review the research process and findings to confirm accuracy and consistency [26, 27, 28].

### 3.7 Confirmability

- **Definition:** A degree of neutrality or the extent to which the findings are shaped by the study participants and not researcher bias, motivation, or interest.

### 3.8 Strategies

◦ Maintaining an audit trail: Documenting all decisions, procedures, and changes made during the research process.
◦ Reflexivity: Continuously reflecting on and documenting the researcher's role and potential biases.
◦ Quoting Participants: Using direct quotes from participants to support findings, demonstrate that they are grounded in the data, and facilitate evaluation of the analysis.
◦ Selecting appropriate data collection methods and obtaining sufficient data to ensure depth and richness.
◦ Emphasizing the importance of selecting meaningful units, coding, and categorizing data accurately.
◦ Providing clear examples of codes, categories, abstraction, and interpretation processes [26, 27, 28].

### 3.9 Ethical Considerations

This study has been submitted to be reviewed by the ethics committee of University of Social Welfare and Rehabilitation Sciences, Tehran, Iran to obtain an ethics approval code (IR.USWR.REC.1403.192). Additionally, the study will be reviewed and approved by the Charité Ethics Committee, Berlin, Germany. Researchers will follow ethical principles, ensuring anonymity, voluntary participation, privacy, confidentiality, and informed consent. Participants' identities will be protected, and data stored securely. Participation is voluntary, with the right to withdraw at any time. Clear explanations of the study's goals, procedures, risks, and benefits will be provided. Privacy will be maintained during interviews. Researchers will be sensitive to participants' needs, especially stroke survivors and their caregivers. Ethical approval and review of any protocol changes will be obtained. Risks will be minimized, and support provided for distressed participants. By adhering to these principles, the study will maintain high ethical standards [26, 27, 28].

## 4 Conclusion

This study protocol outlines the steps for conducting research aimed at developing a directed qualitative content analysis study to identify challenges and facilitators to PPI in stroke research. The detailed, step‐by‐step description of the qualitative research process is intended to guide researchers in conducting studies using similar methodologies. The protocol addresses common limitations faced by researchers working in different languages and discusses solutions to overcome these challenges. Efforts have been made to ensure the quality of the qualitative study, providing criteria that researchers can follow to conduct high‐quality research. Ethical considerations, a primary concern in responsible research, have been meticulously addressed and discussed in detail by the authors. The researchers believe that publishing qualitative research protocols before implementation can pave the way for improved research quality, foster integrated and ethical research practices, and serve as a valuable guide for novice researchers.

## 5 Study Significance/Expected Results

This qualitative study will improve our understanding of involving a diverse range of stroke‐related stakeholders in the research process. It will inform us about operable methods, models, and strategies for PPI applicable throughout the various phases of research. Additionally, it will clarify how the ideas of stroke‐related stakeholders can be synthesized and utilized to shape research design, implementation, and dissemination.

The study will also explore how to effectively work together with stakeholders to improve intervention outcomes, reduce disparities, and sustain tested interventions. The insights gained will help both national and international communities to understand the essential elements required for establishing PPI, and provide a deep understanding of the challenges and facilitators to PPI in stroke research.

Ultimately, the recommendations developed will improve the understanding and participation of all stakeholders engaged in biomedical research in the field of stroke, fostering a more inclusive and effective research environment.

## Author Contributions

**Hamidreza Khankeh:** conceptualization, methodology, supervision, funding acquisition, project administration, writing – review and editing. **Shima Shirozhan:** methodology, data curation, investigation, writing – original draft, writing – review and editing. **Ulrich Dirnagl:** conceptualization, methodology, project administration, writing – review and editing, supervision.

## Ethics Statement

The authors have nothing to report.

## Consent

The authors have nothing to report.

## Conflicts of Interest

The authors declare no conflicts of interest.

## Data Availability

The data that support the findings of this study are available on request from the corresponding author. The data are not publicly available due to privacy or ethical restrictions. The datasets used and/or analyzed during the current study are available from the corresponding author upon reasonable request.
